# Designing for action: adapting and implementing a community-based newborn care package to affect national change in Uganda

**DOI:** 10.3402/gha.v8.24250

**Published:** 2015-03-31

**Authors:** Peter Waiswa, Gertrude Namazzi, Kate Kerber, Stefan Peterson

**Affiliations:** 1 Department of Health Policy, Planning and Management, School of Public Health, College of Health Science, Makerere University, Kampala, Uganda; 2 Global Health, Department of Public Health Sciences, Karolinska Institutet, Stockholm, Sweden; 3 Saving Newborn Lives, Save the Children, Cape Town, South Africa; 4 International Maternal and Child Health Unit, Department of Women's and Children's Health, Uppsala University, Uppsala, Sweden

**Keywords:** newborn health, maternal health, community health worker, pregnancy, postnatal care, Uganda, formative research, health policy

## Abstract

**Background:**

There is a lack of literature on how to adapt new evidence-based interventions for maternal and newborn care into local health systems and policy for rapid scale-up, particularly for community-based interventions in low-income settings. The Uganda Newborn Study (UNEST) was a cluster randomised control trial to test a community-based care package which was rapidly taken up at national level. Understanding this process may help inform other studies looking to design and evaluate with scale-up in mind.

**Objective:**

This study aimed to describe the process of using evidence to design a community-based maternal and newborn care package in rural eastern Uganda, and to determine the dissemination and advocacy approaches used to facilitate rapid policy change and national uptake.

**Design:**

We reviewed UNEST project literature including meeting reports and minutes, supervision reports, and annual and midterm reports. National stakeholders, project and district staff were interviewed regarding their role in the study and perceptions of what contributed to uptake of the package under evaluation. Data related to UNEST formative research, study design, implementation and policy influence were extracted and analysed.

**Results:**

An advisory committee of key players in development of maternal and newborn policies and programmes in Uganda was constituted from many agencies and disciplines. Baseline qualitative and quantitative data collection was done at district, community and facility level to examine applicability of aspects of a proposed newborn care package to the local setting. Data were summarised and presented to stakeholders to adapt the intervention that was ultimately tested. Quarterly monitoring of key activities and events around the interventions were used to further inform implementation. The UNEST training package, home visit schedule and behaviour change counselling materials were incorporated into the national Village Health Team and Integrated Community Case Management packages while the study was ongoing.

**Conclusions:**

Designing interventions for national scale-up requires strategies and planning from the outset. Use of evidence alongside engagement of key stakeholders and targeted advocacy about the burden and potential solutions is important when adapting interventions to local health systems and communities. This approach has the potential to rapidly translate research into policy, but care must be taken not to exceed available evidence while seizing the policy opportunity.

Community-based strategies for maternal and newborn health may prevent up to one-third of the world's annual 2.9 million newborn deaths if universal coverage is achieved (1). A number of community-based trials from Asia demonstrated the potential of task shifting to community health workers (CHWs) for newborn care (2–6), leading to a 2009 Joint Statement on Home Visits for Newborn Care from the World Health Organization (WHO), United Nations Children's Fund (UNICEF) and other partners (7). The recommendations in the Joint Statement were taken up by many countries, including 20 out of 46 countries in sub-Saharan Africa (8). However, few studies have evaluated the implementation effectiveness of home visits for newborn care, and only one study in Ghana has reported on both the process of adaptation (9) and evaluation of the package (10).

Closing the gap from research to practice requires adapting interventions and packages to local settings (11). This is particularly true for interventions that must address strongly held beliefs and practices and tackle complex cultural barriers to care-seeking. There is a lack of guidance in customising interventions for the critical pregnancy, childbirth and postnatal periods. Designing a new package of care requires local contextual information on existing practices and knowledge of the key influentials (12). It is difficult to know in advance how much adaptation of a trial-tested intervention or health system package will be necessary while still maintaining fidelity of the intervention and key elements which play a role in the pathways to behaviour change and ultimately mortality reduction.

To help address this gap in knowledge, the Uganda Newborn Study (UNEST) was designed as a two-arm cluster randomised control trial, taking place in two rural districts in eastern Uganda (13). In intervention, villages volunteer CHWs were trained to identify pregnant women and make five home visits (two during pregnancy and three in the first week after birth) to offer preventive and promotive care and counselling, with extra visits for sick and small newborns to assess and refer. Health facility strengthening was done in all facilities to improve quality of care. Primary outcomes were coverage of key essential newborn care behaviours (breastfeeding, thermal care and cord care). The intervention significantly improved essential newborn care practices, although many key behaviours and practices saw major increases in both arms over the study period. The home visit package was pro-poor, with more women in the poorest quintile visited by a CHW compared to families in the least poor quintile, and more women who delivered at home visited by a CHW after birth compared to those who delivered in a hospital or health facility.

We describe how evidence was used to design and implement this home visit and health facility strengthening strategy for newborn care through UNEST, and how this led to rapid policy and programme uptake, which began influencing scale-up in the country even before the trial was completed. This paper is the second in a series on the UNEST results.

## Design

As a qualitative analysis to determine factors resulting in diffusion and uptake of key elements of the UNEST cluster randomised control trial, we reviewed UNEST internal project literature. This included project reports, meeting minutes, decision trees, supervision reports, and midterm and final reports. In addition, we reviewed and categorised the range of published literature linked to UNEST, including peer-reviewed articles, information brochures, newspaper articles and video documentaries. In-depth interviews were conducted with all members of the UNEST Advisory Committee (*n*=6), conducted by an experienced social scientist with the help of a note-taker. Interviews took place at the respondent's place of work, and focused on understanding current policy, challenges, opportunities and perceptions of stakeholder willingness to adopt Asian models of newborn care.

We also interviewed project and district staff, as well as those involved in newborn care at national level and international experts involved in UNEST design and implementation. Using the WHO and ExpandNet framework on designing projects with scaling-up in mind (14), we analysed the steps and decisions taken based on existing evidence outside Uganda and the local context, particularly in relation to the initial design workshop. Activities, products, key milestones and decisions were plotted on a timeline and analysed thematically.

## Results

The development of the UNEST package followed a linear process, starting with a literature review which helped define the knowledge gaps and local needs. Baseline data collection and formative research were undertaken to describe the current situation in maternal and newborn health and potential barriers to uptake of behaviours and practices. Based on this information, a context-specific package was developed and pilot tested, with refinements made before full implementation. Stakeholder participation at each stage of this process as well as creative engagement of various dissemination outlets enabled rapid adoption and uptake of the package.

### Literature review and package parameters

One of the objectives of UNEST was to adapt India's SEARCH home visit model (2) for use in Uganda. A literature review was done mainly focusing on community newborn interventions, mainly in Asian settings. At the time, the recent release of *The Lancet* Neonatal Series was instrumental in framing the package in terms of the overall burden and causes of death, the survival benefit associated with each intervention, affordability and prioritising community-based care (15, 16). In addition, we explored the Uganda-specific literature and guidelines around maternal and newborn care.

Two major contextual differences were acknowledged arising out of the Ugandan literature. The first was the existence of the historical Village Health Team (VHT) system, which provided a platform for CHW recruitment without the need for creation of a new cadre. The team dynamic of 5–6 members with one focused on maternal and child health was not seen elsewhere. Secondly, dissimilar to the SEARCH model, it was decided that the package would not include sepsis case management at community level, given current clinical guidelines and perceived accessibility of existing curative health services to families. There was considerable discussion and negotiation on this point. The UNEST design phase overlapped with a review of the national Essential Medicines List, which had the potential to include injectable antibiotics for newborn sepsis at the lower-level health facilities, but at the time this intervention was not thought to have sufficient evidence to warrant a significant change in policy.

We also assessed policies and guidelines with regard to community mobilisation and creating demand for maternal, newborn and child health services, drawing heavily on previous work done on breastfeeding counselling and promotion (17–19). There was a notable gap here with a lack of community awareness of and demand for services for preterm and low-birthweight babies. Prior to the UNEST pilot, a network of researchers involved in seven community-based newborn care studies in Ethiopia, Ghana, Malawi, Mozambique, South Africa, Tanzania and Uganda were convened by Save the Children's Saving Newborn Lives programme. The network met in 2006 and 2007 to discuss design issues and formative research, and again in 2009 to review results of the pilot phase and address implementation and monitoring issues. Based on the review of evidence as well as discussions with policy makers and technical experts, it was decided that the package would include preventive home visits in pregnancy and the first week of life using the VHTs linked to the existing district health teams (DHTs).

In order to promote sustainability and potential for replication, policy makers requested that the intervention not include home treatment for neonatal sepsis, community weighing of babies, or taking the baby's temperature. Instead, they recommended strengthening community-facility linkages and referral of sick babies or babies born at home to health facilities.

### Baseline data collection and formative research

Baseline field data collection involved qualitative and quantitative approaches. Formative research built on a similar process and questions used in that underpinning the Newborn Home Intervention Study (Newhints) in the Brong Ahafo region of Ghana (9) (Table 1). Objectives of the formative research were multifaceted, and included:

1. To explore the community's knowledge and perceptions on home care practices, response to neonatal danger signs, care-seeking behaviour and barriers to key practices and care in order to inform behaviour change communication materials and strategies;
2. To investigate health system and socio-economic factors associated with neonatal deaths and the quality of maternal and newborn basic and emergency care in health facilities in order to inform health system strengthening; and
3. To explore the feasibility and acceptability of different supervision and motivation models for CHW visits to pregnant women and newly delivered mothers, and strategies to better link households to health care.

In-depth interviews were conducted with health managers, health workers and community members to understand perceptions of quality and accessibility of maternal and newborn health services and potential barriers and facilitators for behaviour change around key practices.

**Table 1:** Questions to be answered by the formative research and links to intervention design

Aim	Areas of focus	Link to intervention design
Identify gaps in family knowledge and practice of neonatal care	Which priority newborn care behaviours are currently practised at household level and which are not?	Identify priority practices to promote during home visits
Identify causes of newborn death and modifiable factors	What are the biological causes of newborn deaths locally? Which are the social contributors to newborn death? How can these be overcome?	Ensure interventions address the biological causes, social causes and underlying delays related to newborn deaths
Identify whether certain behaviours are more or less likely to be changed	Can the key behaviours be changed? Which resources are available and which are not? Who are the key influencers What are the best channels for facilitating behaviour change?	Identify priority practices for the intervention; identify delivery channels and target audience
Characterise CHWs: availability, role in proposed intervention and their management	Are there CHWs currently? Who are they? How are they selected? What do they do? What caseload is feasible? Can CHWs facilitate problem solving? Are they generally accepted? How can CHWs be motivated?	Guide CHW selection, motivation and supervision
Explore how the intervention logistics can be managed	How can pregnant women be identified and at what stage of pregnancy? Is it possible to visit all women at home during pregnancy and after birth? Is it appropriate for TBAs to serve as CHWs?	Guide identification of primary targets
What is the current quality of maternal and neonatal health services?	Which equipment, supplies and skills are available for neonatal care in health facilities? What causes of death explain the current in-facility mortality?	Guide health facility strengthening and supply-side intervention
Explore how referral can be ensured	Can pregnant women and newly delivered mothers and newborns be referred from community to local health centre when sick? How can linkages with facilities be ensured? What are the current barriers to referral and how can these be overcome?	Guide design of strategies to ensure compliance with referral

Quantitative data collection at baseline involved review of verbal autopsy data from the Iganga-Mayuge Health and Demographic Surveillance Site (HDSS) in order to understand the local causes of newborn deaths and contributing delays (20). In addition, a population-based cross-sectional study was done to establish the baseline levels of maternal and newborn care among 395 newly delivered mothers with babies aged 1–4 months in the HDSS. We assessed the knowledge of maternal and newborn care of 52 health workers using a standardised questionnaire. We also conducted an assessment in 16 health facilities in and surrounding the HDSS to determine the availability of infrastructure, human resources, records, medicines and equipment important for neonatal survival. The facility assessment survey also aimed to identify whether practices and messages were consistent with desired behaviours and whether the quality of care needed to be improved.

The formative findings and baseline data have been published elsewhere (20–23). Table 2 summarises the key findings from the formative research and how this was used to design the intervention. Briefly, some weaknesses were found in terms of both demand for and supply of service, and the linkages between the two were weak. Verbal and social autopsy studies identified major delays at home and in the community associated with stillbirths and neonatal deaths, but also significant limitations in the health facility capacity for newborn care. While community home visits remained the major focus of the trial, based on these gaps a key decision was made to implement and monitor a substantial health systems strengthening component at health facility level. The formative research identified supervision and motivation of CHWs as a critical barrier to address, especially amongst those CHWs previously employed by other projects. Interviews revealed that the CHWs in operation at baseline felt that the work was tiring and time-consuming, and were dissatisfied with the supervision and motivation received for their work (on average 2 days per week).

**Table 2:** Interventions designed to address gaps identified in the formative research

Desired behaviour or practice	Gap identified in formative research	Intervention to address the gap	Context-specific adaptation
Every pregnant woman attends ANC at least 4 times	Only 30% of women had 4 or more ANC visits	CHWs would be trained and supported to identify p. early and provide health education to promote regular ANC attendance	Single-visit ANC coverage already high but extra emphasis placed on importance of multiple visits Extra effort made to identify pregnant women early and track identified vs expected pregnancies to make sure CHWs were capturing women who might otherwise not attend ANC, or would attend late
Every family prepares for birth	Lack of preparation for birth	CHWs would be trained and supported to identify pregnancy early and support family in birth preparation	Lack of materials for birth identified as key barrier for receiving care; mama kits and saving money targeted as key messages during counselling
Every woman delivers in a health facility with a skilled attendant	Only 42% of women delivered from a health facility	CHWs would be trained and supported to identify pregnant women and promote health facility delivery as part of birth preparedness	Increasing demand through CHW visits alone did not seem sufficient to bridge this gap; health facility strengthening component developed largely in response to quality of care concerns at facility
Every woman is aware of maternal and neonatal danger signs	Only 35% of women knew at least 3 maternal danger signs; 48% knew at least 3 newborn danger signs	CHWs would be trained and supported to provide health education regarding maternal and newborn danger signs and care-seeking	Local concepts and terms were used to describe danger signs and to differentiate clinical signs of severe illness from traditional illnesses considered to be severe, e.g. false teeth
Every newborn receives immediate essential care	Babies receiving: Optimal thermal care: 42% Optimal neonatal feeding: 57% Optimal cord care: 38%	CHWs would be trained and supported to counsel on immediate newborn care during pregnancy visits, identify newly delivered mothers and visit them at home on day 1, 3 and 7 and support postnatal care	Given the high facility delivery rate, the strategy to reach mothers and babies on day 1 focused on home deliveries with an attempt to connect the health facility and CHW with information that the pair had been discharged
Every woman and baby receives quality care on a continuum from home to health facility, and back	Of those babies that died, 50% were related to delays at home, and 30% were related to delays in receiving care at health facilities Only 30% of health workers had knowledge of newborn care Health facilities lacked equipment, drugs and supplies for newborn care	CHWs would be trained and supported to identify newly delivered mothers, support postnatal care, screen for danger signs and facilitate referral Health facilities strengthened through training, provision of basic drugs and equipment, and maternal and perinatal audit introduced	A referral slip given to the mother by the CHW was introduced to counteract the long waiting times at health facilities in this setting. When health workers were sensitised about these referral cards they were more likely to see the mother-baby pair quickly The mix of public and private facilities required a unified approach with both sectors targeted with health facility strengthening interventions

ANC=antenatal care.

### Intervention package design and pilot

The formative research findings were presented at an intervention design workshop in Jinja, eastern Uganda, in September 2008. The primary goal for the intervention design workshop was to apply the gathered evidence to ensure that the designed package, and its components were applicable and appropriate in the context of rural eastern Uganda; that the intervention was consistent with national policies; and that it would be feasible and sustainable. We therefore invited to the workshop the following: representatives from national and regional levels of the Ministry of Health (the key neonatal policy makers and programme coordinators), the two DHTs of Iganga and Mayuge districts where the study was to be done, and non-governmental organisations and United Nations agencies as well as experts in neonatal health, behaviour change communication and those working with community volunteers. Technical assistance for the design workshop was provided by a researcher from the Newhints study in Ghana. Findings from the formative research were presented to invited stakeholders. In addition, the *a priori* UNEST design as per submitted funding protocol, which included piloting the use of community injectable antibiotics and provision of supplies such as thermometers and weighing scales to CHWs, were also presented and discussed. Stakeholders were split into three groups to discuss the community component; health facility strengthening; and the monitoring and evaluation framework. Each group had a facilitator, and they discussed until a consensus was reached. The groups presented conclusions in plenary, and again facilitated discussions were held until consensus was reached.

The CHW package is shown in Table 3. In line with existing Ugandan guidelines in policy but not routinely implemented, CHWs were recruited from the village or parish, trained for 1 week, and supervised by existing health workers from the nearest public sector health unit. In total, 61 CHWs were selected by their communities with the aim of identifying individuals with empathy, experience of similar problems and situations, who were respected in the local community, and considered to be a natural helper or someone that community members would naturally go to in the event of a problem. Female CHWs were preferred although males were also accepted. More details, including the selection, training and supervision of CHWs as well as the content of each visit, can be found elsewhere (13, 24).

**Table 3:** Timing and content of CHW home visit package

Home visits during pregnancy	
1st home visit Timing: First trimester or as early as possible	Negotiation for ANC, at least 4 ANC visits at health facility Birth preparedness; prepare for a health facility delivery with mother and family members Screen for danger signs and facilitate referral Counsel on family planning Health education
2nd home visit Timing: Third trimester, 7–8 months’ gestation	Reinforce birth preparedness Encourage delivery in health facility Counselling on: Maternal and newborn danger signs Family planning Immediate newborn care practices (optimal feeding practices, hygienic cord care, thermal protection)
Postnatal home visits 3rd home visit Timing: Within 24 hours after delivery, or as soon after discharge from the facility as possible	Screen for and counsel on maternal and newborn danger signs, facilitate referral in case of danger signs Counsel on and demonstrate thermal care (skin-to-skin, wrapping and infrequent bathing) Counsel on exclusive breastfeeding including attachment and positioning. If the mother chooses not to breastfeed, the CHW should determine why and support accordingly Counsel on and demonstrate hygienic practices including hand washing and clean cord care Counsel on ITN use, good nutrition, rest for mother and family planning Refer for immunisation (if applicable) and facilitate birth registration
4th home visit Timing: Third day after delivery	Follow-up on issues noted from previous visits Screen for maternal and newborn danger signs and if present facilitate referral Counselling as per previous visit
5th home visit Timing: Seventh day after delivery	Follow-up on issues noted from previous visits Screen for maternal and newborn danger signs and if present facilitate referral Counselling as per previous visit Promote access to under-five clinics and family planning at 6 weeks
Sick and/or small babies Two additional home visits	Follow-up to ensure compliance with referral, or identify reasons why additional care was not sought Provide counselling on extra care for sick and/or small babies, particularly kangaroo mother care

Based on the key messages arising from the formative research and agreed upon at the design workshop, a set of pictorial counselling cards on maternal and newborn health was developed to engage family members. A card with pictorial reminders of the main messages and a space to record the date of the next visit and the CHWs’ contact details was developed, to be left with the family after the first home visit. The family was also provided a template for a birth- preparedness plan for use during counselling. A danger sign screening card was developed to facilitate easier identification of sick newborn babies, as well as a referral card to encourage prompt care-seeking, with space to record the reason for referral in order to assist the health workers, as well as a feedback note to the CHW to be filled in by the health worker who attended to the sick newborn.

CHWs were to be given a set of materials to facilitate their work, and provide credibility and motivation. This included an identity card; a bag for materials; a t-shirt; notebook; counselling and screening cards; referral forms; registers; birth-preparedness forms as well as cards to leave with families. They would also be provided with report forms for monthly reports and a ‘mama kit’ (clean delivery kit) for demonstration to mothers on the key requirements needed for delivery, which the women could purchase at the clinic or through community sellers.

The issue of payment and supervision of CHWs was amongst the biggest decisions made by the UNEST team. In the past, VHTs were not paid by the Government but were dependent on project funds for remuneration, resulting in a non-holistic VHT service. At the same time, non-payment was not deemed acceptable by current and potential CHWs. Through discussions with the national stakeholders working on the VHT strategy as well as international experts engaging in similar work, a decision was taken that all CHWs be paid a token allowance, labelled a transport allowance, to the amount of 10,000 Ugandan shillings (about US$ 5 at the time of the study) monthly, to be given at supervision meetings or refresher trainings. Supervision of CHWs in other projects in Uganda ranged from weekly (mainly in drug distribution trials or projects) to quarterly (as per the Government mandate). It was felt by health workers, CHWs and the study team that less frequent supervision left the CHWs demoralised, but weekly supervision was unsustainable without project-specific staff. Given that the supervisors comprised parish mobilisers, subcounty inspectors and health workers from nearby clinics who had other tasks in their daily remit, a monthly group supervision meeting and directly-observed supervision visits were put in place. As CHWs became more comfortable in their role, supervision was stepped down to take place on a quarterly basis. This strategy has been found to be effective in imparting and retaining skills (25, 26).

The CHW training used materials adapted for Uganda from the regional workshop on Home-Based Care for Mothers and Newborns organised by UNICEF Eastern and Southern African Region in Nairobi, Kenya. The training was conducted by a team of Ugandan health professionals who had attended the training of trainers course, with involvement of DHT staff from the two districts. Many of the trainers were involved in developing training materials and the design workshop. The training for CHWs was skills-based, and focused on promoting key selected practices from formative research and the recommendations of the workshop. While the VHT strategy requires CHWs to be living in the community in which they serve, this carried a potential risk of CHWs not being seen as qualified to give health advice, especially on such a sensitive thematic area as maternal and newborn care – typically the purview of elder women in the family and traditional birth attendants (TBAs). In order to solidify community acceptability, following training all CHWs were to be commissioned as *omuwi w'amagezi*, literally meaning a person who gives health advice.

The health systems strengthening component of the trial was also identified. In addition to home visits by CHWs during pregnancy and after delivery, interventions for demand creation included sensitisation of community leaders and TBAs to the role of the CHWs and the importance of skilled care during childbirth. To address issues of supply, health managers and administrators of private clinics and pharmacies would be engaged and informed of the new home visit package. Health workers in all facilities with a reasonable maternity caseload (15–20 per month) would be given a once-off training in newborn care. In addition, it was decided that basic equipment and supplies (e.g. partographs, weighing scale, neonatal Ambu bag and masks for resuscitation) and a catalytic, once-off supply of drugs for newborn care were to be provided to health facilities. Full details of the intervention are described elsewhere (13, 24).

Following the intervention design workshop, meetings to adapt the UNICEF materials were held with support from the Ministry of Health. Other materials of reference came from the Ministry of Health, WHO, Ghana and Malawi; these were scrutinised, analysed, modified and adapted to the local rural Ugandan context. Guided by a principle of designing randomised control trials, a decision was taken that the pilot study would be conducted in Ibulanku subcounty outside the HDSS in order to avoid later-stage contamination. Based on results of the pilot, the package was further refined.

### Implementation of the package, stakeholder engagement and national uptake

Following the pilot, CHWs were recruited and trained within the HDSS. They started home visits with only counselling cards and forms. Later the CHW bags, inclusive of the demonstration mama kit, were obtained and distributed to each CHW. The chairmen of each village in which CHWs resided were invited to the deployment meeting. Results of in-depth interviews with local council leaders, facility-based health workers and families identified that CHWs were highly appreciated in the community and seen as important contributors to maternal and newborn health at grassroots level (27). CHWs themselves reported being highly motivated to do their work, arising out of the fact that they were selected by and trained in the community and provided a service which linked to the formal health system. Intrinsic motivators (e.g. community appreciation and the prestige of being ‘a doctor’), monetary (such as a small transport allowance) and material incentives (e.g. bicycles, bags) were important motivators to varying degrees.

There was engagement of policymakers and decision leaders from village to national level. Several forms of engaging were used, including meetings, brochures, newspaper publications, television interviews, documentary videos, scientific publications, presentations at conferences, and field visits. These efforts were based on resources and relationships already existing within the study team, but new relationships were made as necessary to ensure that key decision-makers were informed. Reports and materials were regularly shared with key stakeholders. At the district level, an implementation committee consisting of representatives from the two DHTs and research team met regularly throughout the design and pilot phase to make operational plans about community entry, sensitisation and selection of CHWs, training, supervision, the motivation system for CHWs and other intervention activities. The committee also looked at the proposed workloads for CHWs to assess how feasible they were and how they compared with volunteer CHWs in other settings with similar training. Using a standardised tool developed specifically to assess financial and human costs of newborn care services, time spent by the CHW on home visits, activities related to the programme, and other health-promotion activities was assessed through a self-reported survey of 18 randomly selected CHWs (29% of total CHWs in the intervention villages). Similarly, seven supervisors (38% of total supervisors in the intervention villages) were randomly selected and asked to record time spent on supervision. The relatively low average time spent per home visit (82 min) and number of visits per week (1.5) were used as advocacy messages to demonstrate to DHTs and national policymakers that home visits are a feasible addition and do not constitute a massive time commitment on the part of the volunteers.

The multidisciplinary advisory body to the Ministry of Health on newborn health issues, the National Newborn Steering Committee (NNSC), established in 2006, provided an obvious platform for sharing information, receiving critical feedback and learning about similar efforts under way in other areas of the country. The NNSC undertook a field visit to UNEST to learn more about the home visit package as well as to provide technical input to project staff and the implementation committee. The implementation committee meetings as well as NNSC discussions were used as an opportunity to gain consensus on key decisions and to vet products as well as identify next steps in the design process. Workshops involving external technical experts further aided this process.

At the start of the UNEST implementation the Ministry of Health was also developing guidelines, materials and policies for revitalisation of the national VHT strategy, which called for 5–6 CHWs in each village with 1–2 devoted to maternal, newborn and child health activities. Additionally, the national adaptation of integrated community care management (ICCM) of childhood illness was ongoing, but was only capturing services for children aged 2–59 months. The UNEST team took advantage of this opportunity to share experiences and materials. As a result, the national VHT and ICCM packages include the home visit schedule, key messages around newborn care, and UNEST behaviour change communication materials. These packages are being scaled-up for nationwide use at community level. The facility-based training materials on newborn care have also been used in trainings around the country and incorporated into the national service standards for newborn care (28). Finally, a documentary video highlighting newborn care within UNEST was adopted by the Ministry of Health to be used as an advocacy tool at meetings and on national television.

## Discussion

UNEST achieved significant improvements in birth preparedness and the essential newborn care practices, including breastfeeding, hygienic cord care and thermal protection – practices that are associated with reduced neonatal mortality. The general improvement in these and other practices and service coverage across both the intervention and control areas may be explained at least in part by the health facility strengthening which impacted both trial arms, but also by the secular trend towards improved maternal and newborn care. CHWs who were selected by their communities with district-led training and supervision were able to identify and visit almost all the pregnant women, especially those from the poorest families and those who delivered at home or with TBAs.

This analysis shows how evidence was used to adapt new interventions proven elsewhere into the Uganda health system policy and programme context, and how this led not only to an adapted intervention but also to rapid policy adaptation and scale-up of components of UNEST while the study was still going on. Key success factors included identifying high-impact best practices, rigorous formative research and pilot testing to develop a package that fits the local context, wide stakeholder engagement and involvement, and taking advantage of a policy window with consistent messages. These findings may have implications for how interventions found to be effective in other contexts can be adapted into local policies and programmes. However, caution is required so as not to oversell the effectiveness of a package prior to availability of local evidence.

Evidence from outside of Uganda, particularly the Asian community-based newborn care trials, were extremely influential in increasing attention for the overlooked burden of newborn mortality in the country. Policy guidelines and training materials from UNICEF, the WHO and others on newborn care were also widely disseminated. The third paper in *The Lancet* Neonatal Series – which greatly influenced UNEST and newborn care in Uganda overall – presents a framework for systematically scaling-up newborn care that has been followed closely (Panel 1 ) (29). While other countries focused primarily on strengthening community level platforms (30), the decision to systematically address community and facility issues in UNEST has set the stage for newborn health programming nationwide. The decision for CHWs to undertake a preventive/promotive health package and not treat neonatal sepsis makes sense in this context, but perhaps limits the scope of impact of these workers. Identifying newborns with danger signs and ensuring prompt care-seeking was challenged by the routine visit schedule (i.e. passive case-finding). Compliance with referral for sick newborns was thought to be high, but also associated with the perceived quality of care at the nearest health facility and outside the control of the CHW (27). This is also an issue where CHWs provide curative services; preventive and promotive messages given through routine counselling visits may not receive as high priority (31).

Panel 1 **Steps to scale-up neonatal health**

Step 1: Assess the situation and create a policy environment conducive to neonatal health

Step 2: Achieve optimum neonatal care within the constraints of the situation

- Start with outreach or family-community care if the health system is not strong
- Identify and address missed opportunities within the formal healthcare system
- Coordinate across programmes relevant to neonatal health

Step 3: Systematically scale-up neonatal care

- Strengthen supply
- Improve demand
- Overcome supply and demand obstacles

Step 4: Monitor coverage and measure effect and cost

The formative research together with the quantitative baseline data collection identified key knowledge gaps and informed an iterative process to design a package of interventions specifically tailored to the context at the time. Experienced researchers, including external technical assistance were required for this component building on similar formative research in Ghana (9) as well as Nepal (32). Triangulation of results with findings from other assessments in Uganda identified some of the differences in the local settings and would be advisable, although not essential, upon introduction in other districts.

The decision to engage a wide group of stakeholders from the outset allowed for increased ownership and the ability to elicit expert experience. These stakeholders participated in designing the intervention, behaviour change materials and health worker training materials. The engagement of the NNSC was particularly strategic given their prominent role in newborn health policies and programming (33). In the absence of such a body, substantially more effort would have been required at national level to rally interested or potentially interested partners and to join up other efforts working on the same or similar goals through health systems strengthening and/or VHTs, for example, ICCM of childhood illness. One challenge encountered with district-level stakeholders outside the study area was the concern that the intervention might not be replicable given the study site within the HDSS. At the local level involvement of the DHTs and health facility staff in monitoring and supervision is likely to have increased sustainability of the intervention, and having DHTs involved in dissemination and speaking in various fora provided a sense that the package was not wholly led by researchers but owned by the whole community. The intervention has since been taken up in six nearby districts.

The success of UNEST in rapidly influencing policy could be attributed firstly to the stakeholders’ active engagement, but also to the existing policy window for home-based preventive and promotive newborn care. At the time there was demand from national-level stakeholders and external pressure to develop effective low-cost interventions to reduce neonatal mortality in order to accelerate efforts towards achieving Millennium Development Goal 4. Thus, in designing UNEST there was an *a priori* goal of designing an intervention that would inform policy and lead to rapid scale-up. The remit was expanded somewhat following the baseline findings, so that it also allowed for health systems strengthening and addressing low quality of facility-based care. However, major decisions were taken which limited the intensity of the intervention in order to keep it in line with national policy guidelines. Both CHW and health worker trainings were no more than a week in duration, and CHW supervision was by health workers from nearby health facilities rather than UNEST project staff. Also in line with existing guidelines, the CHWs were not paid and instead were given transport refunds and other small tokens as incentives.

Despite the rigorous design and pilot phase, ongoing process monitoring and, to a certain degree, adaptation is also important. For instance, CHWs preferred doing pregnancy home visits as opposed to postnatal visits in the first few days after birth. When supervisors were provided this information, they were able to adjust guidance, leading to sustained improvements in both pregnancy and postnatal home visits. Another challenge was that families and CHWs were not adequately identifying small babies, given that CHWs were not given a weighing scale because of the prohibitive cost. A separate study was conducted to design and validate a foot length card that CHWs carry with them and use during the first postnatal visit. The foot length card has been taken up for use within the national VHT roll-out (34). Where these package adaptations occur in a study setting, particularly a randomised design, this makes the intervention an evolving one, which needs to be taken into account in trial design and reporting.

The uptake of elements of the UNEST package prior to completion of the trial reflects the success of this approach, but also the favourable policy window for both newborn care at both facility and community levels. Researchers should be careful not to oversell the intervention and to identify which elements may be specific to evaluation of the package and which are necessary for expansion. Additionally, there should be scope to re-evaluate the package when results are available. The cost of scaling-up the package to both the health system and families is also important to consider.

## Conclusions

Interventions proven elsewhere and recommended by international experts require local adaptation if they are to fit into local health systems, be accepted by the community and taken to national scale. Understanding local epidemiology, sociocultural context and barriers to seeking care can play an important role in designing an integrated package of health system improvements. The use of evidence combined with local testing and engagement of key stakeholders at community, district and national level was critical to adapting and testing the UNEST intervention package that has been taken up widely and integrated into broader community-based and facility-level packages of care.
